# A Digital Patient-Led Hospital Checklist for Enhancing Safety in Cataract Surgery: Qualitative Study

**DOI:** 10.2196/periop.9463

**Published:** 2018-07-16

**Authors:** Aline C Stolk-Vos, Jolet JE van der Steen, Constance HC Drossaert, Annemarie Braakman-Jansen, Bart LM Zijlmans, Leonieke W Kranenburg, Dirk F de Korne

**Affiliations:** ^1^ Rotterdam Ophthalmic Institute Rotterdam Netherlands; ^2^ Section Health Services, Management and Organisation Erasmus School of Health Policy and Management Erasmus University Rotterdam Rotterdam Netherlands; ^3^ Section Medical Psychology and Psychotherapy Department of Psychiatry Erasmus Medical Center Rotterdam Netherlands; ^4^ Dutch Federation of Patients Utrecht Netherlands; ^5^ Vakgroep Psychologie, Gezondheid & Technologie University of Twente Enschede Netherlands; ^6^ Rotterdam Eye Hospital Rotterdam Netherlands; ^7^ Duke-NUS Medical School Singapore Singapore; ^8^ KK Women’s & Children’s Hospital SingHealth Duke-NUS Academic Medical Centre Singapore Singapore

**Keywords:** patient participation, checklist, cataract, surgery, patient safety, handheld computers, health information management, health communication, information technology

## Abstract

**Background:**

Surgery holds high risk for iatrogenic patient harm. Correct and sufficient communication and information during the surgical process is a root solution for preventing patient harm. Information technology may substantially contribute to engaging patients in this process.

**Objective:**

To explore the feasibility of a digital patient-led checklist for cataract surgery, we evaluated the experiences of patients and nurses who have used this novel tool with a focus on use, appreciation, and impact.

**Methods:**

A multidisciplinary team, including cataract surgeons, nurses, pharmacists and administrative representatives developed a 19-item digital patient-led checklist for cataract patients who underwent surgery in an ambulatory setting. This “EYEpad” checklist was distributed to patients and their companions during their hospital visit via an application on a tablet. It contained necessary information the patient should have received before or during the surgical preparation (8 items), before anesthesia (2 items), and before discharge (9 items). Patients and their companions were invited to actively indicate the information they received, or information discussed with them, by ticking on the EYEpad. Our qualitative research design included semi-structured individual interviews with 17 patients and a focus group involving 6 nurses. The transcripts were analyzed by 2 independent coders using both deductive and inductive coding.

**Results:**

All but one of the 17 patients used the EYEpad, occasionally assisted by his or her companion (usually the partner). In several cases, the checklist was completed by the companion. Most patients felt positively about the usability of the EYEpad. Yet, for most of the patients, it was not clear why they received the checklist. Only 4 of them indicated that they understood that the EYEpad was used to determine if there were sufficient and correct information discussed or checked by the nurses. Although most nurses agreed the EYEpad was easy to use and could be a useful tool for improving patient engagement for improving safety, they felt that not all elderly patients were willing or capable of using it and it interfered with the existing surgical process. They also anticipated the need to spend more time explaining the purpose and use of the EYEpad.

**Conclusions:**

Our results showed that a digital patient-led checklist is a potentially valid way to increase patient participation in safety improvement efforts, even among elderly patients. It also illustrates the crucial role nurses play in the implementation and diffusion of technological innovations. Increased patient participation will only improve safety when both healthcare workers and patients feel empowered to share responsibility and balance their power.

## Introduction

Health care delivery is too often not “free from accidental injuries,” according to the Institute of Medicine definition of patient safety [1]. In Dutch hospitals, about 2.6% patients die and 1.6% are harmed annually due to preventable, unnecessary actions [2]. The associated costs are estimated at 0.5% of the national hospital care budget and, since only direct costs were considered, this calculation is likely an underestimation of the real costs [2].

Surgery is a high-risk area for iatrogenic patient harm [3,4]. Iatrogenic harm is the unintended or unnecessary harm or suffering arising from any aspect of the health care delivery besides the patient’s condition [3]. Errors that cause iatrogenic harm to patients should be mitigated before they can cause harm [3].

The last decade has seen increasing awareness and focus on patient safety [5-9]. Traditionally, patient safety has been viewed as the sole responsibility of health professionals with patients as passive recipients. Nowadays, patient participation is increasingly being recognized as a key component in the improvement of health care since, in contrast to health care staff, patients are around during all steps of the care pathway [10-13]. However, few studies show patients as active participants in safety efforts, and these studies mostly focus on listening well and speaking up when concerned [14-17].

Communication between patients and professionals is a major issue in safety [18]. The handover of information from professional to patient is critical for successful recovery after surgery and compliance with postsurgical instructions [19]. Studies have shown that a lack of communication between patients and professionals in surgical care resulted in less optimal outcomes [18,20]. Insufficient and contradictory postsurgical information on health status and patient behavior requests are major safety issues.

Although it is known that communication of the “right things” at the “right moment” is important for preventing iatrogenic patient harm, it is difficult to optimize this process because patients are concerned with many things during their care pathways. Information technology may substantially contribute to engaging patients in activities to improve patient safety [21,22].

To increase patient participation in enhancing safe care, we developed an online checklist called the EYEpad for cataract patients to be used during their admission. Cataract surgery involves removal of opaque lens and replacement with an implanted artificial intraocular lens (IOL) is the most frequently performed surgery in the world [23]. The feasibility of this checklist—in terms of utilization, appreciation, and impact—according to patients and nurses has not yet been determined. To explore the feasibility of the digital checklist for cataract surgery, we evaluated the experiences of patients and nurses who have used the checklist at the Rotterdam Eye Hospital in the Netherlands.

## Methods

### Design

We used a qualitative approach to explore patients’ and nurses’ experiences with the digital EYEpad checklist. The definition of semistructured interviews by Green and Thorogood is “In a semistructured interview, the researcher sets the agenda in terms of the topics covered but the interviewee’s responses determine the kinds of information produced about those topics, and the relative importance of them” [24]. At appointments, we conducted semistructured interviews with patients and their companions, and a focus group with nurses.

### Setting

Participants were recruited at the Rotterdam Eye Hospital, the only eye hospital in the Netherlands providing secondary and tertiary eye care. The hospital has a specialized ambulatory cataract pathway where about 6500 cataract surgeries are performed annually.

### Intervention: The EYEpad

**Figure 1:**
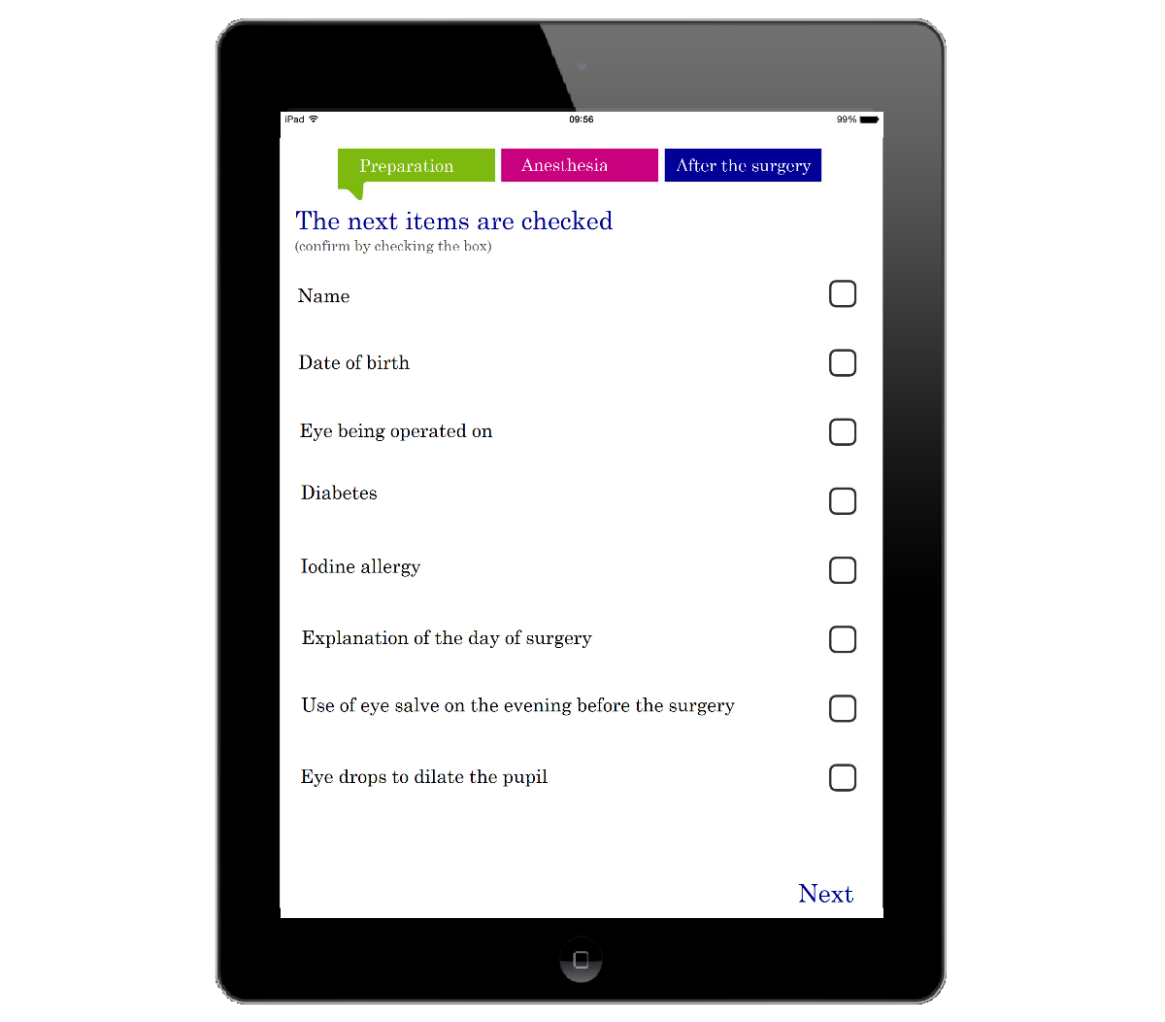
Overview of the first subchecklist (preparation), containing information on specific events in the care pathway.

Patients often had questions about how to care for their treated eyes after discharge from the hospital. To prevent this, initially a paper card was designed to relay information to patients before their discharges. The card served as a memory aid for nurses to inform patients about these points, but was rarely used. Subsequently, a checklist for patients was designed. A multidisciplinary team, including cataract surgeons, nurses, pharmacists, and administrative representatives developed a 19-item patient-led checklist for cataract patients who underwent surgery in an ambulatory setting. The items were based on a review of nurses’ current, often inconsistent, and not formally acknowledged, check moments. An initial gross-list of more than 30 items was reduced to 19, all of which were agreed upon by the multidisciplinary team. The checklist was first tested on paper by patients and later modernized into an application for the tablet called EYEpad. This checklist was distributed to patients and their companions via the application EYEpad, on a tablet, during their hospital visits. It contained 3 lists with necessary information the patient should have received during three contact moments with medical professionals on the day of their surgery: before or during surgical preparation (8 items), before anesthesia (2 items), and before discharge (9 items; see Figure 1 for the screenshot of the subchecklist and Textbox 1 for all 19 items).

The EYEpad is handed to the patient on the day of the surgery. The patient is supposed to indicate whether the predefined information on the checklist was discussed with the nurse. Based on this checklist, the patient is expected to address the nurse regarding the missing items. The checklist is also used by the nurse, to confirm whether all information has been addressed in a consistent manner. While the nurse checks the list, he or she can provide missing information for the patient and perform a formal acknowledgment (“check”). Finally, the patient can add his or her own questions to ensure that these questions are addressed during the dismissal conversation. Patients could also use the tablet for other general, educational, or entertainment functions, such as news services and games.

### Participants

#### Patients

During a period of two weeks, patients who were scheduled first and last in the morning and in the afternoon were approached to participate in the study. A registered nurse recruited participants according to the following inclusion criteria: (1) age older than 18 years, (2) first cataract surgery, (3) ability to understand Dutch, (4) absence of severe comorbidities, and (5) absence of mental or cognitive disorders.

The selected patients were approached by phone one day before their hospital visit. Patients were given information about the study and asked whether they wished to participate. It was emphasized that participation was voluntary and their decision about participating in the study would have no effect on their treatment. When patients agreed to participate, the researcher fixed a time for a short interview at the hospital immediately after the patient’s discharge.

**Overview of the 19-item checklist.**

**I Preparation phase (8 items):**

- Patient name
- Patient date of birth
- Eye to be operated on
- Diabetes status
- Iodine allergy status
- Explanation on day of surgery proceedings
- Explanation on eye balm application on eve of surgery
- Explanation of dilatation drops

**II Anesthesia phase (2 items):**

- Time-out
- Anesthetic eye drops

**III Postsurgical phase (9 items):**

- Review of surgical proceedings
- Pain assessment
- Postsurgical patient flyer
- Availability and application of eye drops at home
- Postsurgical telephone review (date and time)
- Removal of eye bandage
- Checking of pupil size and form
- Photo surgical team
- Eye drop application information and training

#### Nurses

The ambulatory surgical center (ASC) manager invited all nurses to a regular department meeting and allowed the researcher to use a part of the meeting for a focus group.

### Measurements

#### Participant Interviews

Prior to the interview, the participants provided informed consent for participation and for tape recording of the interview. All interviews were conducted by a trained psychologist (JVDS). Interviews took an average of 10 minutes and took place in a separate room, behind closed doors, to preserve the patient’s privacy.

During the interviews, an interview guide with 24 open-ended questions, derived from published literature and in consultation with staff members of the hospital and the University of Twente, was used. The interview questions focused on (1) EYEpad utilization: “Did you use the EYEpad?”; (2) appreciation of the EYEpad: “What did you like/dislike in the EYEpad?”; and (3) impact of the EYEpad: “What does feeling safe in a hospital mean to you?” Patients were asked explicitly to motivate and support their answers. All interviews were audiotaped. Ethical approval was obtained from the ethics committee of the University of Twente (#13196).

#### Focus Group with Nurses

Prior to the focus group session, the nurses were asked to complete a 10-item questionnaire. This questionnaire was intended to stimulate the participants to think about the topics discussed during the focus group. We chose this approach to prevent group thinking by the participants.

All nurses were asked to provide consent for participation and tape recording of the focus group. The focus group lasted 60 minutes. During the focus group session, a script with open-ended questions, derived from published literature and in consultation with staff members of the hospital and the University of Twente, was used. The questions focused, as they did during the patient interviews, on (1) EYEpad utilization: “What instructions did you give to patients during handover of the EYEpad?”; (2) appreciation of the EYEpad: “What do you consider to be positive and negative aspects of the EYEpad?”; and (3) impact of the EYEpad: “What do you consider as benefits of the EYEpad?”. Finally, the focus group addressed (4) the future of the EYEpad: “What needs to be changed for sustainable use of the EYEpad?” The minutes of the meeting were included in the analysis.

### Data Analysis

The audiotaped data from the interviews was transcribed verbatim. Transcripts were deductively coded into one of the three main categories: utilization, appreciation, and perceived impact. Next, the fragments in each category were further divided into subcategories, using inductive analysis, meaning the categories were inferred from the data, rather than from the existing literature. Coding was conducted by 2 coders (JVDS, AS). Differences were discussed until a consensus was achieved [24].

## Results

### Description of Participants

From the 32 selected patients, 19 patients met the inclusion criteria. Seventeen patients accepted the invitation to participate in the study. Two patients refused to participate because they did not feel well enough to be interviewed after their surgeries. Eleven out of 17 (65%) patients were female. The average age was 69 years, ranging from 58 to 88. Almost all patients were accompanied by their partner (n=12) and others by their daughter (n=2), son (n=1), or another relative (n=1). One patient was not accompanied by a companion.

Six of the 18 registered nurses participated in the focus group. All nurses were female (n=6). The average age of the nurses was 46 years, ranging from 20 to 57. Most nurses (n=4) worked at the ASC for at least 5 years.

#### Description of Themes

Three themes emerged from the data analysis: utilization, appreciation and impact. The subthemes that belong to these themes can be found in Multimedia Appendix 1.

#### Utilization of the EYEpad

##### Patients

All but one patient used the EYEpad. In several cases (n=9), the EYEpad checklist was completed by the companion as the patients could not clearly see because their eyes were dilated or their reading glasses were stored in a locker. The companions only entered the patients’ answers.

I have completed it, but yes, actually I have only pressed the buttons. You have completed the answers.

Companion 3

Three patients completed the EYEpad on their own because they were more familiar with a tablet than their companion was. One patient and his companion did not use the EYEpad because they were not familiar with the use of a tablet device.

Most did not completely understand why they received the EYEpad. They took the EYEpad without further enquiries and assumed it was part of the hospital administration or it was for quality improvement. In only four cases, respondents indicated that the EYEpad was meant to validate if all necessary information was given to the patients and if nurses checked important information for patients’ safety.

[Silence] No, I assume things automatically; they need to know who you are, and they repeat that often. [Silence] I think that’s part of the administration.

Patient 1

Besides using the EYEpad application, patients and their companions could use other functionalities on the tablet, like the web browser, playing a game, or watching movies. One patient read the news on the internet, to relax, before her surgery.

Yes, I have checked the news that was available, so I had something to read...That killed the waiting time, so I enjoyed it.

Patient 17

The others did not use the other functionalities on the tablet because they were unfamiliar with tablet functions or did not feel a need for it.

…And I was afraid that there was just one application [the EYEpad app], so I thought, yes [laughs], keep it like this, it is functioning well now, and I should not peddle someone else’s tablet.

Patient 2

During the surgery, I have watched the [intraocular live viewing] monitor, so you don’t need the iPad [tablet] at that time.

Companion 3

Fourteen participants, who already knew how to use a tablet, reported that the EYEpad was easy to use. However, some difficulties were experienced. First, some respondents reported that it was hard to fill in their birth date because the scroll menu moved fast. Second, some respondents did not understand the jargon used in the EYEpad, for example, “*time-out*.” Further, it was not clear to all respondents when to use specific checklist tabs, considering there were 3 different tabs. Lastly, one respondent reported there was little time between the nurses’ explanation and the use of the EYEpad; this patient did not have sufficient time to open the EYEpad and get used to it before he had to use it.

Yes, especially for that age, it was taking an aim.

Companion 2

Yes, that’s right [laughing], a kind of roulette, as it kept on rotating.

Patient 2

When asked whether the participants preferred the version of the EYEpad on the tablet or on paper, 14 participants preferred the digital version because of the usability and ability to save data.

It is easy to complete, briefly touch and a checkmark appears.

Patient 11

Two participants preferred a paper questionnaire above a tablet; both reported low eHealth literacy. Both participants were females. One female was aged 82 and was accompanied by her daughter, and the other female was 72 years old and accompanied by her partner.

##### Nurses

Five of the 6 nurses provided the EYEpad to all patients, except when the patient did not have a companion or when the patient was very old. However, during the focus group, there was a discussion as to whether a patient could be “too old”.

When someone is very old, I don’t offer him an EYEpad.

Nurse

But some elderly are very good with tablets, so I think that’s no reason to not give it to an elderly patient.

Nurse

Yes, indeed, some elderly are able to handle the EYEpad, and like it very much, so age should not be a discriminator.

Nurse

Some see you approaching them with the piece, and they don’t look very happy.

Nurse

Another reason for not providing the EYEpad was the workload experienced by the nurses. The nurses were unanimous that the EYEpad was subordinate to their primary work: treating patients.

Sometimes you need to assist a colleague, or sometimes you are very busy, or something else needs your attention; the EYEpad is then the first to neglect or skip.

Nurse

Five nurses mentioned that they found it hard to give the correct explanation when they provided the EYEpad to a patient. This was caused by different reasons. They referred to the busy schedule and the number of patients around during the provision of the EYEpad. Also, the perceived patient knowledge of the tablet played a role.

If it’s very busy, it’s difficult to give a proper explanation. You have less time.

Nurse

If the patient is familiar with an iPad, the instructions can be done fast because you don’t have to explain how an iPad works.

Nurse

Although nurses were generally positive about the EYEpad usability, they noticed, just like the patients, a few difficulties. First, it was not clear which action was related to the term “*anesthetic drops* ” on the second tab of the checklist.

I just still do not understand fully what must be ticked at “anesthetic drops.” Is that the moment that we tell the patient they receive anesthetic drops and show which ones? Or is it the moment that we give the anesthetic drops?

Nurse

Second, it was unclear why only “iodine allergy” was in the checklist because other allergies of the patients were also important to know. Finally, they noticed it was not always easy to go back to the top of the checklist when the checklist was finished.

#### Appreciation of the EYEpad

##### Patients

The EYEpad was well appreciated by patients and companions. Most of the respondents reviewed the EYEpad as *“good”* or *“fine”* (n=12). They especially appreciated the checkpoints. Both patients and companions indicated they felt more involved in the health care process on using the EYEpad.

Well, I thought it is an extra check, for you can’t check these things (eg, eye to be operated on) often enough.

Patient 2

We now need to think ourselves, and that was, ehh, you are more involved at least.

Companion 13

##### Nurses

Most nurses did not appreciate the EYEpad for two reasons. The first reason was that the EYEpad caused some agitation for both patients and nurses. According to the nurses, some elderly patients were scared they had to use a tablet. Agitation was also experienced when the patients’ companions moved from the waiting room to the preparation room to complete the second checklist.

What I have experienced as troublesome is that the companions of the patients now more often move to the preparation room taking their entire possessions, because they have to complete over there a second list. This is not really the intention and creates a lot of agitation.

Nurse

Yes, indeed, previously the companion came just along to the preparation [room] as a patient had some degree of anxiety, but now they all come in to complete the checklist.

Nurse

The second reason why not all nurses appreciated the EYEpad was because it was time-consuming.

The provision and explanation of the EYEpad still just takes a lot of extra time. It is not always the case that you are there with a short explanation, because most of the patients have several questions about it, such as how it exactly works.

Nurse

They also mentioned positive aspects of the EYEpad. First, the use of the EYEpad improved the reputation of the day center.

It seems luxurious and very modern.

Nurse

Second, they thought it was nice that younger patients were fine with the EYEpad. Third, they were generally positive about the usability. Most of the nurses (n=4) mentioned that the EYEpad was easy to use. Two mentioned that, although they were not completely familiar with a tablet, they always resolved it together with a colleague.

I think it is sometimes still quite a bit of a search, even though I know how an iPad works, but fortunately,you will always bring it to an end.

Nurse

#### Impact of the EYEpad

##### Patients

Most patients saw no safety benefits associated with using the EYEpad (n=10). They did not know the purpose of the EYEpad. Six patients, however, thought the EYEpad could contribute to safety because of all the extra data checks.

Uh, that the EYEpad would help for safer care, here, in the hospital? Well, no, I really don’t see that link directly.

Patient 5

Yes, that would be possible, I think, or yes, I do not really know. What do you [companion] think?

Patient 10

Yes, you know, it certainly can, as long as the nurse still take[s] care of those points [unchecked items on the checklists].

Patient 4

##### Nurses

According to the nurses, the contribution of the EYEpad to safety is not yet known. They felt time they spent on the EYEpad was too short to evaluate its contribution to patient safety. They were still uncomfortable with the EYEpad and felt their explanation to the patients was still suboptimal. The nurses named several impact factors associated with the EYEpad. First, they reported that the EYEpad had a positive influence on the empowerment of patients. The patients were more involved in their care process, more alert, and more conscious of their own responsibility.

The patient is more involved in his or her surgery process by the EYEpad.

Nurse

By the EYEpad the patient becomes more alert and sees more things during the care process.

Nurse

With the EYEpad you make the patient and his or her companion more aware of their own responsibility.

Nurse

Second, the EYEpad had a positive impact on the patients’ companions because the companions could use the other functionalities of the tablet to relax. Further, the EYEpad influenced the interaction between patients in a positive manner.

If a patient does not understand the EYEpad or encounters a problem, patients help each other. This creates more contact between patient and companion. Perhaps this also reduces the patient’s anxiety.

Nurse

Next, the EYEpad could have a negative impact on patients and their companions because they could get distracted by the EYEpad during intake, get surprised following presentation of the EYEpad, and companions could feel obligated to the patient.

If you give the tablet, people go straight to work with the tablet, therefore people pay less attention to the nurse.

Nurse

Patients do not yet know anything about the EYEpad when they arrive at the day center on the day of their surgery. It can overwhelm them.

Nurse

Some patients often do not dare to say they do not like it [tablet], because we [nurses] offer them from the hospital, and therefore they think that it is obligatory.

Nurse

Companions are more concerned with the iPad [tablet] than with the patient, making the guidance or support falls away.

Nurse

Lastly, the EYEpad may worsen the supportive role of the companion if the companion gives more attention to the tablet than to the patient.

## Discussion

### Principal Findings

This study showed that the use of the EYEpad as a digital patient-led checklist in cataract surgery is feasible. Feasibility has been demonstrated in three ways. First, the EYEpad was well appreciated by patients. Patients were positive about the additional checks and felt more involved in their care processes. Second, we found the EYEpad, beside some practical difficulties, was easy for patients and nurses to use. Third, we found that the EYEpad helped patients feel empowered.

However, there remains room for improvement. The EYEpad, with its current instructions, can increase nursing workload. Furthermore, an improved introduction on the rationale and use of the digital checklist is needed because the purpose of the EYEpad was not always clear to the participants. Improved instructions are likely to further enhance patient experience, increasing patients’ abilities to understand and influence their own care. This was suboptimal in this study because some patients participated just because the EYEpad was handed to them and not because they were motivated to use it.

### Limitations

This study had several limitations. One limitation was that we held just one focus group with nurses. More focus groups could have yielded more information about the nurses’ viewpoints. Moreover, participation in the focus groups was voluntary and in the end, only 6 of the 18 nurses participated. The nurses who participated likely held viewpoints that differed from nurses who did not participate.

Another limitation was that the purpose of the EYEpad was not clear to all patients and nurses at the start of the study. Only 4 patients indicated they understood the purpose of the EYEpad. This may be due to the limited explanation the nurses gave about the checklist. Apparently, a more elaborate explanation is needed to better understand the purpose of the EYEpad, both for patients and nurses. Previous work has suggested the success of checklist implementation largely depends on a clear explanation of the “why” and “how” [22]. A better understanding of the “why” in this study could further improve the feasibility.

Further, although we inquired as to patients’ general experiences with the EYEpad; we did not explicitly address electronic health (eHealth) or health literacy during this study. Therefore we cannot be sure that needs related to EYEpad use were specifically addressed.

### Comparison with Prior Work

In our study, the checklist was patient-led instead of team-led. We found this helped to empower the patients in their own care pathways. We suggested two possible explanations for why patients felt more empowered by using the EYEpad. First, patients may feel more engaged in their own care process. Using health technology makes patients feel more involved in their own care [25]. As indicated by Horwitz and Greysen et al, knowledge alone is not sufficient for proper self-care after surgery [26,27]. Hospitals need to facilitate a good transition, and recovery at home will improve, if patients and caregivers jointly explore patient-centered strategies.

Aujoulat et al described the success factors for patient empowerment. They found that the basics of patient empowerment were to provide reassurance and opportunities for self-exploration on how to manage illness [14]. Second, patients may experience a smaller gap between care professional and patient, which could help them discuss personal questions or issues that may interfere with their treatment.

Besides empowerment, we also found that the EYEpad increased patient participation. Checklists are supplementary tools that encourage critical thinking and conversation [22]. The EYEpad may help patients engage in their own care. It can ease barriers to preventing harm—for example, not speaking up in the case of suspected errors. A study has shown that communication problems are the root causes of wrong IOL implants in cataract surgery [28]. In New York State, wrong implant-related errors account for 63% of the total number of malpractice claims, and data from Veterans Health Administration showed that approximately half of surgical errors were attributed to the use of the wrong implant [29]. Increased patient empowerment and participation using the checklist can prevent IOL-related errors and thereby improve patient safety.

A surprising finding was that nurses experienced the checklist as “extra work” instead of as a supportive tool for their daily tasks. This may be because the goal of the checklist was not clearly explained. Furthermore, not all nurses were involved in the development of the checklist, which may have made them feel less engaged.

### Learning Points

Before further development of the EYEpad, some hurdles should be addressed. These include providing clear instruction on the rationale for the professionals involved and an improved introduction and explanation of the purpose of the checklist for patients. Communication about the objective of the new digital technology, both with health care staff and patients, is a vital element for successful implementation. It is important to include nurses and other health care professionals from the early idea generation stage, into development and iteration, to generate support and interest. Communication about the objective of the EYEpad must be clear, both to nurses and to patients. Further, our study showed that the practical implication involved listening closely to the care pathway: Which moments are best for the digital EYEpad checklist to be distributed given the planning of the surgical treatment flow? In the current process the use of the EYEpad sometimes disrupted the existing flow, when it should have contributed to a smoother and high-quality care process.

After these hurdles have been considered, the EYEpad can be further developed and implemented. We found that the EYEpad could encourage learning, for example by conscious information acquisition by patients. We did not give specific attention to eHealth and health literacy of participants. More attention to eHealth and health literacy may improve the level of learning.

Further, the checklist should relate to the various steps of the current care process. The better the checklist is implemented, the more structural value it will add toward patient participation in enhancing safe care. It would be useful to make a connection between the checklist and the patients’ records to give the professionals insight into the data in a more accessible way. In addition, future studies should make a connection between the checklist and other patient tools to give patients a more complete overview of their care process.

### Conclusion

In conclusion, we showed that a digital patient-led checklist during surgery was a feasible instrument in cataract care. Our findings suggest that a digital checklist could increase health literacy and provide enhanced guidance on the day of surgery. Our results also demonstrated the crucial role nurses play in the logistics of technological innovations. Increased patient participation will only improve safety as both health professionals and patients feel empowered to share responsibility and balance power.

## Appendix

Multimedia Appendix 1 Themes and subthemes.

Multimedia Appendix 2 19-item checklist (in Dutch).

## Abbreviations

ASC: ambulatory surgical center
eHealth: electronic health
IOL: intraocular lens
