# Supplementary material for: A Digital Patient-Led Hospital Checklist for Enhancing Safety in Cataract Surgery: Qualitative Study
Source: JMIR Perioper Med. 2018 Jul 16;1(2):e3. doi: 10.2196/periop.9463 (PMC7709842; doi:10.2196/periop.9463)
Supplement: Multimedia Appendix 1 [file periop_v1i2e3_app1.pdf]

Appendix I Themes and subthemes

1. Utilization
   - Completed by patient / companion
   - Acceptation of EYEpad
   - Use of other functionalities
   - Experienced difficulties
   - Paper or tablet
2. Appreciation
   - Positive points
   - Negative points
3. Impact
   - Safety in healthcare
   - Goal of EYEpad
   - EYEpad and safety
