# Supplementary material for: A Digital Patient-Led Hospital Checklist for Enhancing Safety in Cataract Surgery: Qualitative Study
Source: JMIR Perioper Med. 2018 Jul 16;1(2):e3. doi: 10.2196/periop.9463 (PMC7709842; doi:10.2196/periop.9463)
Supplement: Multimedia Appendix 2 [file periop_v1i2e3_app2.pdf]

Multimedia Appendix – 19 item checklist

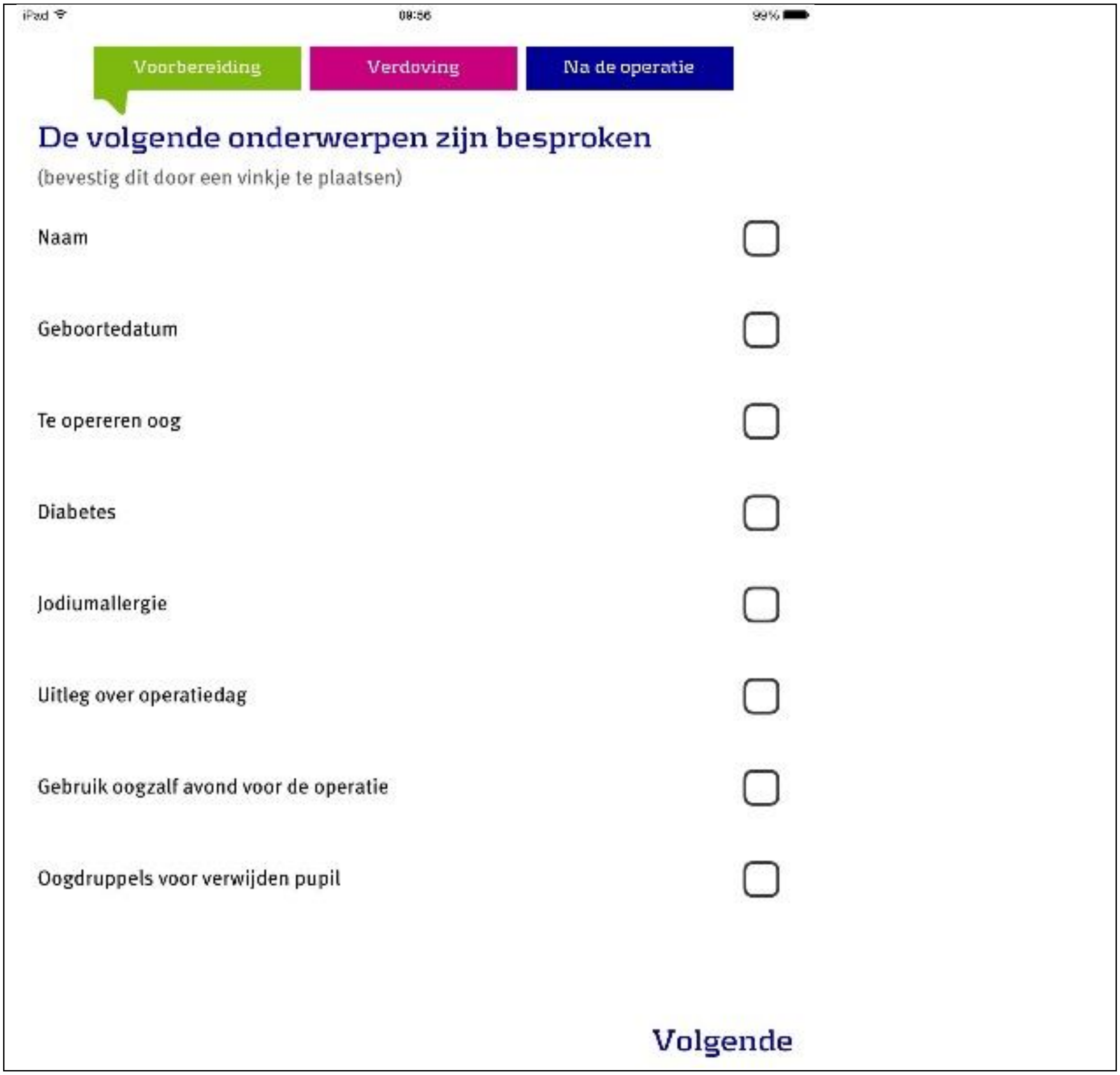

iPad 09:56 99%

Voorbereiding | Verdoving | Na de operatie

De volgende onderwerpen zijn besproken

(bevestig dit door een vinkje te plaatsen)

Time-out ☐

Verdovingsdruppels ☐

Vorige Volgende

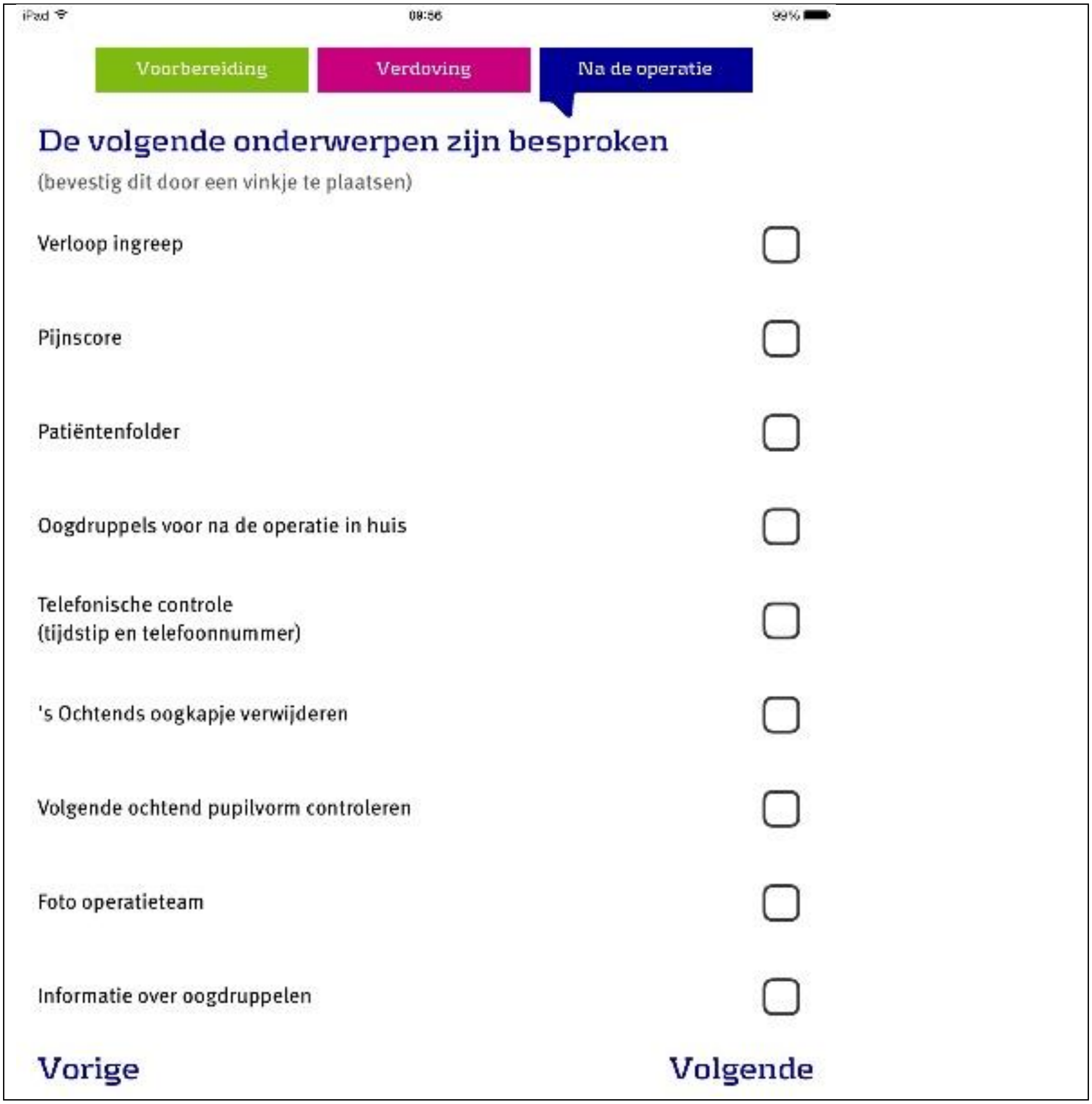
iPad 09:56 99%

Voorbereiding | Verdoving | Na de operatie

De volgende onderwerpen zijn besproken

(bevestig dit door een vinkje te plaatsen)

- [ ] Verloop ingreep
- [ ] Pijnscore
- [ ] Patiëntenfolder
- [ ] Oogdruppels voor na de operatie in huis
- [ ] Telefonische controle (tijdstip en telefoonnummer)
- [ ] 's Ochtends oogkapje verwijderen
- [ ] Volgende ochtend pupilvorm controleren
- [ ] Foto operatieteam
- [ ] Informatie over oogdruppelen

Vorige | Volgende
